# How can we better frame COVID-19 public health messages?

**DOI:** 10.1007/s44202-022-00042-6

**Published:** 2022-06-13

**Authors:** Enoch Teye-Kwadjo

**Affiliations:** 1 Department of Psychology, University of Ghana, Legon, P. O. Box LG84, Accra, Ghana; 2 Department of Industrial Psychology, Stellenbosch University, Private Bag X1, Matieland, 7602 South Africa

**Keywords:** Fear appeal, Message framing, Public health campaigns, COVID-19

## Abstract

This Perspective discusses the use of fear appeals in promoting health behaviour. The discussion establishes that fear appeal-based public health messages (i.e. public health messages that emphasise the consequences of failing to engage in a particular health behaviour) have two components (1) perceived threat and (2) perceived efficacy. A perceived threat has two subcomponents (a) perceived high susceptibility (e.g. ‘I can contract COVID-19’) and (b) perceived high severity (e.g. ‘COVID-19 can kill me’). In a parallel fashion, perceived efficacy has two subcomponents (a) high response efficacy (e.g. ‘Staying at home can reduce my risk for COVID-19’) and (b) high self-efficacy (e.g. ‘I can stay at home’). This discussion demonstrates that for fear appeals to have a desirable effect on health behaviour change, all of the four conditions (i.e. high perceived susceptibility, high perceived severity, high response efficacy, and high self-efficacy) are important and need to be fulfilled. However, empirical evidence shows that the four conditions are almost never fulfilled, calling into question the effectiveness of using fear appeals in promoting health behaviour change. In contrast, gain-framed public health messages (i.e. public health messages that highlight the benefits of engaging in a particular health behaviour), which do not require the fulfillment of these four conditions, have been shown to have positive effects on behaviour change outcomes. We argue that public health messages that highlight the benefits of engaging in COVID-19 preventive behaviour can have persuasive, desirable effects on health behaviour change, compared to public health messages that highlight the consequences of failing to engage in a particular COVID-19 preventive behaviour.

## Introduction

At the start of the COVID-19 pandemic, the World Health Organisation (WHO) called on the citizens of all nations to spread facts and solidarity instead of fear and panic [1]. While this wise counsel from the WHO seemed to have been adopted fairly well worldwide, there were however instances of the spread of fear and panic in some countries [2, 3]. Other researchers, working independently, have warned against circulating COVID-19 fear-based health messages on social media and in the general public because they have the potential to backfire [4–7]. There is evidence that fear-arousing public health messages can give rise to fear contagion with detrimental impacts on mental health [8–11]. “Fear appeals are persuasive messages designed to scare people by describing the terrible things that will happen to them if they do not do what the message recommends.” ([12]; p.329). We ask, where does the weight of existing fear appeal research evidence leave us on COVID-19 preventive behaviour promotion?

## Mixed results from fear appeals research

The use of fear appeals in public health communication has largely produced mixed results [13, 14], with researchers disagreeing on its appropriateness or lack thereof. Whereas some researchers have reported that fear appeals (also referred to as loss-framed appeals) do not bring about desirable changes in health behaviour [15], and that the prospect theory inspired risk-framing hypothesis underpinning fear appeals has important theoretical flaws [16], other researchers have reported contrary results [17]. Specifically, opponents of the use of fear appeals in health promotion point to the inconsistent empirical findings that suggest both linear and curvilinear relationships between fear and persuasion as well as the ethical concerns that arise when health promotion campaigners deliberately evoke fear in order to persuade people to adopt a health behaviour [18, 19]. Those who support this view posit that gain-framed messages are much more effective, compared with loss-framed messages [20–22]. For example, a meta-analysis of 93 studies involving 21,656 participants demonstrated that in an infectious disease prevention, gain-framed public health appeals that highlight gains or advantages of complying with the recommended behaviour change inherent in the message, were more effective and persuasive than were loss-framed public health appeals [23].

Moreover, another meta-analytic work involving 189 effect sizes, polled from 94 studies demonstrated that gain-framed public health appeals were more effective at preventing health threats than were loss-framed public health appeals regarding health behaviours such as smoking cessation and skin cancer prevention [24]. Other work found that, gain-framed public health appeals had stronger, persuasive effects on a person’s intentions to quit smoking than did loss-framed public health appeals [22, 25].

In contrast, proponents of the use of fear appeals argue that when used with caution and under the correct circumstances, fear-arousing public health messages can be effective [26, 27]. For example, Tannenbaum and collegues conducted a meta-analysis involving 127 fear appeal studies, comprising 27,372 participants to examine the effectiveness of fear appeals in health-promoting behaviour change [28]. They found a positive relationship between fear appeals and attitudes, intentions, and behaviour. However, the authors observed that the fear appeal—behaviour change relationship was moderated by factors such as high response efficacy, high perceived severity, and high perceived susceptibility, with the moderating variables interacting with fear appeal messages to have an enhancing effect on behaviour change [28]. We turn to the importance of these factors below.

## Emerging consensus against the use of fear appeals

Health behaviour researchers have recently started building a consensus regarding the appropriateness of fear appeals in public health promotion. Ruiter and colleagues reviewed the sixty-year old fear appeal literature to summarise the current state of the research evidence relative to fear appeal’s effectiveness in public health campaigns [29]. They concluded that public health messages designed to increase perceived response efficacy and high self-efficacy had greater protective effects on health behaviour change than were threatening public health messages designed to arouse fear and to heighten risk perception [29]. For similar meta-analytic research conclusions, see the following studies [30–32].

Additionally, meta-analytic evidence demonstrates that the mixed findings regarding the role of fear appeals in health promotion arise primarily from misinterpreting the extant empirical evidence and theory [33]. This review work shows that fear appeal-based public health messages may have positive effect on behaviour change only under specific, rare circumstances. According to the study, these circumstances are ‘perceived threat’ and ‘perceived efficacy’ on the part of the recipient of a fear-arousing message. The review concludes that for fear appeals to have their desired effect on behaviour change, a recipient of a fear-arousing message ought to perceive that they are susceptible to the health threat [33], namely [a] perceived high susceptibility (i.e. the perception that one is susceptible to the stated health threat e.g. ‘I can contract COVID-19’), and that the health threat would affect them severely, should it come to pass, namely [b] perceived high severity (i.e. the perception that the stated threat would be severe in scope and magnitude e.g. ‘COVID-19 can kill me’). In addition, the recipient ought to possess [c] high response efficacy (i.e. a person’s beliefs about whether the recommended behaviour change would be effective against the health threat e.g. ‘Staying at home can reduce my risk for COVID-19’) and [d] high self-efficacy (i.e. a person’s beliefs about their confidence to carry out the recommended health behaviour change e.g. ‘I can stay at home’), because in the presence of low response efficacy and low self-efficacy, fear-arousing public health messages weaken into non-significance and have the ability to bring about defensive reactions, instead. These meta-analytic results are consistent with those of previous research which found that response efficacy was necessary for fear appeal to have effect on recommended behaviour change [34]. Correspondingly, using peer commentaries, various health behaviour researchers, working independently, have corroborated the results of the meta-analysis by pointing out the ineffectiveness of fear appeals in health promotion campaigns [35–38].

Further, earlier research has reported similar findings, but which results seemed to have been ignored or have largely remained inaccessible to public health professionals [27, 39, 40]. The World Health Organisation’s Outbreak Communication Guidelines and the U.S. Centers for Disease Control and Prevention’s [CDC] (2020) Crisis and Emergency Risk Communication (CERC) manual also require that in a health crisis, such as the on-going COVID-19 pandemic, public health messages should be reassuring, empathetic, and respectful [41–43]. Thus, these manuals seem to support the view that gain-framed public health messages are desirable during a health crisis situation.

## Conclusion

The COVID-19 pandemic has brought into sharp focus the importance of effective message framing for public health communication. There is considerable empirical evidence that fear-arousing public health messages rarely have desirable effects on behaviour change. Correspondingly, despite their widespread use, growing research evidence seems to question the effectiveness and relevance of fear appeals in health promotion. In contrast, empirical findings and meta-analytic results have demonstrated that gain-framed health messages can have persuasive, desirable effects on behaviour change. The seeming convergence in the fear appeal literature is encouraging, as it provides sound theoretical explanations regarding the processes associated with health message acceptance and rejection. Of note is the acknowledgement that various factors can strengthen or weaken the fear-persuasion relationship [20, 21, 28, 44]. For example, Jeong and colleagues found that the effect of fear appeals on attitudes toward smoking cessation depends on a person’s level of anger [44]. Other researchers also found that the effect of fear appeals on attitudes, intention, and behaviour is moderated by response efficacy and its constituent components of susceptibility and severity as well as gender [28]. These statistical mediation and moderation results reflect a major criticism of the positive linear relationship view, namely, that fear is held to associate positively with health message acceptance (i.e. that higher levels of fear arousal have greater persuasive effects on health behaviour change minus the effect of intervening variables). The converging literature elucidates when and why fear appeals fail by indicating that there are processes that moderate or mediate the effects of message framing on behaviour [45].

Taken together, growing research evidence (i.e. the weight of existing research evidence) seems to provide substantial support for the use of gain-framed public health campaigns in general, suggesting an answer to our research question. From the foregoing, we argue that public health campaigns that highlight the benefits of engaging in COVID-19 preventive behaviour (gain-frame) can have greater persuasive and desirable effects on health behaviour change than public health campaigns that emphasise the consequences of failing to engage in COVID-19 preventive behaviour (fear appeal/loss-frame). In other words, we can better frame COVID-19 public health messages by highlighting the benefits people stand to gain when they engage in a particular behaviour such as wearing nose masks. Nevertheless, more research is required to clarify the situation (i.e. general health promotion situation versus crisis health promotion situation such as a pandemic situation) in which gain-framed public health messages are most effective.
